# Immuno-Oncology Biomarkers for Personalized Immunotherapy in Breast Cancer

**DOI:** 10.3389/fcell.2020.00162

**Published:** 2020-03-17

**Authors:** Vida Vafaizadeh, Zeinab Barekati

**Affiliations:** Department of Biomedicine, University of Basel, Basel, Switzerland

**Keywords:** immunotherapy, biomarker, breast cancer, immune checkpoint blockade, anti-PD-1 and anti-PD-L1

## Abstract

The immune checkpoint blockade therapy has drastically advanced treatment of different types of cancer over the past few years. Female breast cancer is the second leading cause of death in the overall burden of cancers worldwide that is encouraging healthcare professionals to improve cancer care management. The checkpoint blockade therapies combined with novel agents become the recent focus of various clinical trials in breast cancer. However, identification of the patients who are responsive to these therapeutic strategies remained as a major issue for enhancing the efficacy of these treatments. This highlights the unmet need in discovery and development of novel biomarkers to add predictive values for prosperous personalized medicine. In this review we summarize the advances done in the era of biomarker studies and highlight their link in supporting breast cancer immunotherapy.

## Introduction

Recent advances in cancer therapy present immunotherapy as a prospect change in treating various cancers. The immune-checkpoint blockade (ICB), designated as a cutting edge therapy, is used in increasing number of advance cancer diseases with durable responses compared to most chemotherapy and targeted therapies (Ansell et al., 2015; Gettinger et al., 2015; Larkin et al., 2015; Spencer et al., 2016; Tray et al., 2018). Breast cancer is the most common malignancies among women worldwide and many breast cancers have been recently determined immunogenic and enriched in tumor-infiltrating lymphocytes (TILs) (Cimino-Mathews et al., 2016).

The ICB monotherapy, anti-programmed death-1/programmed death-ligand-1 (anti-PD1/PD-L1), has demonstrated promising outcomes in metastatic triple negative breast cancer (Nanda et al., 2016; Schmid et al., 2018; Adams et al., 2019a, b; Emens et al., 2019). There is a considerable attention for developing immunotherapy-based strategies to escalate anti-cancer responses and to reduce the side effects, such as trials on combination therapy of anti-PD-1/PD-L1 with chemotherapy agents or combination with targeted therapies in metastatic patients (Nolan et al., 2017; Domchek et al., 2018; Nicolas et al., 2018). Moreover, developing strategies on combining different ICBs are appealing in breast cancer treatment, such as combining anti-PD-1/PD-L1 with anti-cytotoxic T-lymphocyte associated protein-4 (anti-CTLA-4) (Wolchok et al., 2017) or other co-inhibitory molecules (Chester et al., 2018; Harding et al., 2019). One of the major challenges in this regard is to establish predictive biomarkers for the stratification of breast cancer patients benefiting from these ICBs therapeutic strategies.

In March 2019, the U.S. Food and Drug Administration (FDA) approved the first ICB drug, a PD-L1 antibody called Atezolizumab (Tecentriq) in combination with a chemotherapy agent, for the treatment of triple-negative metastatic breast cancer patients (TNBC) (NCT02425891) (Schmid et al., 2018; Emens et al., 2019). The TNBC is a subtype of the disease with frequency of 15% and lacks hormone receptors, estrogen receptor (ER), progesterone receptor (PR), and human epidermal growth factor receptor (HER2) (Anders et al., 2016). The Atezolizumab authorized to be applied only on metastatic TNBC patients whose tumors express the PD-L1 protein that is characterized by an FDA-approved test, VENTANA PD-L1 (SP142), as a predictive biomarker. This was a significant fundamental step in predicting clinical benefit of only one ICBs combination strategies in breast cancer treatments. However, different ICBs agents and strategies are going to bring new treatment modalities for this disease. Therefore, there is an unmet need in developing novel predictive biomarkers for proper selection of patients who are benefiting from ICBs treatments and for avoiding unnecessary toxicity in unresponsive patients. Furthermore, identifying predictive biomarkers are necessary for better management of the expensive health care costs, especially for those patients that are unlikely to be responsive to the ICBs therapies. Here we summarize the attempts that have been done on the discovery of major predictive biomarkers in liquid biopsies, tumor tissues and tumor microenvironment that might contribute into advancing prediction of therapeutic decisions as well as the future challenges in this era.

## Liquid Biopsies Biomarkers

Over the past few years, considerable effort has been done in discovery and development of liquid biopsy-based biomarkers, as it is minimally invasive, cost effective and can be replicated during patients’ follow-up (Wan et al., 2017; Pantel and Alix-Panabieres, 2019; Rothwell et al., 2019). These biomarkers can be detected in blood, cerebrospinal fluid and urine of cancer patients but not in healthy individuals. Today, liquid biopsy-based biomarkers are defined as soluble proteins, exosomes or other vesicles transmitting proteins or nucleic acids driven from a tumor, circulating tumor cells (CTCs) and circulating tumor DNAs (ctDNAs) (Kohler et al., 2011; Siravegna et al., 2017). All these properties make liquid biopsy-based biomarkers attractive in immunotherapy for the assessment of predictive biomarkers at the baseline or in monitoring therapy response.

The plasma proteins such as soluble PD-L1, cytokines and exosomes’ bound proteins are considered as important source of information in biomarker discovery and development. A high level of soluble PD-L1 has been demonstrated a poor prognosis in ICB response (Okuma et al., 2018). Nonetheless, the plasma level of soluble PD-L1 can be increased due to the different physiological conditions and diseases e.g., pregnancy or autoimmune diseases (Yanaba et al., 2016; Jovanovic et al., 2018). Therefore, the utility of soluble PD-L1 as a cancer biomarker remained as a controversial issue that need more in depth studies to define proper cut-off to differentiate between cancer therapeutic response and other diseases or physiological conditions. The cytokine and chemokine signature in cancer indicated potential predictive value in ICB therapy (Arrieta et al., 2017). Development of a cytokine panel to evaluate ICB response for patient classification in breast cancer seems to be encouraging, for instance, it has been shown that IL-27 up-regulated PD-L1 and promoted breast cancer growth (Yan et al., 2019), yet more studies need to be done to develop a proper panel of cytokines with predictive values in breast cancer. Another plasma protein candidate biomarkers are exosomes, a detective level of RNA molecules and proteins including PDL-1 are packed in exosome and secreted from cancer cells into the blood and lymphatic systems. Exosomes’ transmitting PD-L1 can bind to PD-1 on T-lymphocytes and consequently inactivate immune system from attacking cancer cells (Yang et al., 2018; Poggio et al., 2019). Increased level of circulating exosomal PD-L1 is a predictive marker for patient clinical response, e.g., indicating poor prognosis in melanoma patients (Chen et al., 2018). Moreover, as it was mentioned, exosomes and other vesicles contain detective levels of different classes of RNA molecules including, protein-coding RNAs (mRNA) and non-coding RNAs (e.g., miRNA) (Umu et al., 2018). Recent studies demonstrated that miRNAs directly or indirectly regulate the expression of different immune checkpoints on T-cells and on Tumor cells or APCs (Zhang et al., 2019). Hence, miRNAs that specifically control one-target checkpoints are favored in biomarker development. Various miRNAs such as miR-34a, miR-17-5p, miR-15b, miR-193a-3p, miR-197miR-200c showed correlation with expression of PD-L1 in tumor tissues as well as in sera or plasmas, and purposed to have predictive values in ICBs therapy of different cancers (Chen et al., 2014; Cortez et al., 2016; Ahn et al., 2017; Audrito et al., 2017; Kao et al., 2017; Fan et al., 2019). In breast cancer, a panel of thirteen miRNAs has been identified that directly target and down-regulate B7-H3. Among these thirteen miRNAs, expression of miR-29 is associated with higher survival rate of breast cancer patients (Nygren et al., 2014). Therefore, circulating exosomal biomarkers are considerably perceived as a robust source of information – both proteins and nucleic acids e.g., miRNA – to be investigated in breast cancer (Meng et al., 2019).

Circulating tumor cells are considered as important source of liquid biopsy-based biomarkers because they are driven from different sites of a tumor and could provide with more information about overall tumor characteristic. In breast cancer CTC clusters are recognized as a valuable prognostic biomarker and are associated with increasing metastatic potential (Aceto et al., 2014). Importantly, CTCs can interact with neutrophils and form CTC-neutrophil clusters that are proliferative and highly efficient metastatic precursors in breast cancer (Szczerba et al., 2019). The first study on expression of PD-L1 on CTCs was reported in patients with metastatic breast carcinoma (Mazel et al., 2015), and later it was investigated in other types of cancer. Further study on CTC/PD-L1 indicated that the frequency of PD-L1 positive CTCs are significantly higher in metastatic breast cancer patients compared to non-metastatic patients (Schott et al., 2017). These findings suggested CTC/PD-L1 assay as a potential non-invasive marker for stratification of patients benefiting from anti-PD-L1 therapies in clinical trials (Mazel et al., 2015; Schott et al., 2017). The expression of another important immune checkpoint member, B7-H3, on CTCs of breast cancer patients has been also reported. The B7-H3 positive CTCs showed pronounced correlation with Ki-67 expression, a tumor proliferation marker, and were proposed to be a potential biomarker or target for immune checkpoint blockade therapies in breast cancer (Pizon et al., 2018). CTCs are valuable source of tumor information to develop new biomarkers, however, there are some challenges on the reproducibility of the results which are linked to the applying various techniques for isolation and enriching of these cells. The techniques should be standardized among health service laboratories with the aim to provide qualified analysis of both the epithelial CTCs and the cells undergoing epithelial to mesenchymal transition (EMT), and at the same time to minimize contaminations with other cells such as circulating macrophages with the same surface marker expression. This will enable investigators to establish the CTC biomarkers with predictive or prognostic value in immune therapy.

The ctDNAs have been for decades center of attentions as a crucial source of liquid biopsy-based biomarkers. Today advances in the ctDNAs extraction and enrichments combined with next-generation sequencing (NGS) have contributed to development of valuable biomarkers such as genome instability number (GIN) (Jensen et al., 2019), tumor mutational burden (TMB) and aberrant DNA methylation (Sina et al., 2018) for cancer immunotherapy response stratification of patients.

Different attempts on identification of ctDNA GIN have indicated some merit in therapeutic decision-making (Ahlborn et al., 2019) and in monitoring of breast cancer recurrence (Yang et al., 2019). Nevertheless, the genome-wide analysis of GIN using ctDNA demonstrated GIN dynamic changes upon ICB therapy that enabled monitoring treatment outcome in cancer patients (Jensen et al., 2019). On the contrary, analysis of GIN using ctDNA marked no predictive value in response to ICB therapy for breast cancer (Jensen et al., 2019). Nevertheless, this study was done on a small breast cancer cohort; therefore, to have a meaningful closure about the GIN clinical values, the larger clinical trials should be conducted in breast cancer patients. The TMB assessment in tumor tissues is considered as a promising biomarker, solo or in combination with PD-L1 immunohistochemistry (IHC), with predictive value in immunotherapy efficacy in various types of cancer (Danilova et al., 2016; Ready et al., 2019; Samstein et al., 2019). The utility of ctDNA for assessment of TMBs delivers support for limited or inaccessible tissue samples to improve therapeutic decisions for some cancers (Gandara et al., 2018); however, this is highly dependent on the selection of the gene-targeted panel to evaluate TMBs, and most probably needs to be customized for each cancer type. In addition, the efficiency and precision of the assay using ctDNA to depict TMB is dependent on high coverage of the assay (Gandara et al., 2018; Georgiadis et al., 2019; Pasini and Ulivi, 2019). For breast cancer a NGS panel of mutations associated with 76 target genes, MammaSeq^TM^, have been recently developed with applicable use of ctDNA. MammaSeq^TM^ showed encouraging result in detection of somatic mutations and monitoring disease burden. However, the assay has limitation in capturing all known mutations associated with cancer and is not specific for rare events in ctDNA (Smith et al., 2019).

DNA methylation changes are key to the development and progression of certain cancers (Chatterjee et al., 2018). Aberrant DNA methylation signature demonstrated great potential to be used as a ctDNA biomarker in cancer (Barekati et al., 2010; Radpour et al., 2011; Sina et al., 2018). It has been shown that DNA hypermethylation of promoter or distal enhancer regions play role in low expression of PD-L1 (Y. Zhang et al., 2018) and demethylation of PDCD1 promoter activates PD-1 expression (Mishra and Verma, 2018). Treating cancer cells with the DNA methyltransferase (DNMT) inhibitors resulted in a better response of anti-CTLA-4 immunotherapy (Chiappinelli et al., 2015). Moreover, other studies indicated that the expression of PD-L1 on cancer cells is associated with global hypomethylation that could play a role in the regulation of PD-L1 expression. This information is emphasizing on potential indication of DNA methylation signatures as biomarkers, which might suggest additional treatments or combination therapies to modulate responsiveness to PD-1 inhibitor treatment (Emran et al., 2019). Furthermore, the genome-wide technology and their corresponding data analysis illustrated a signature that might enable guiding the prediction of ICB immunotherapy response in cancer (Duruisseaux et al., 2018; Guo et al., 2019). However, relatively little attention has been given to develop a customized panel of genomic regions with aberrant methylation patterns for breast cancer.

## Tumor Tissue Biomarkers

A limited number of tumor biomarkers were already assessed in clinical trials of the ICBs approved by the FDA (Table 1). In tumor cells both genomic and non-genomic factors are studied as potential biomarkers to predict the response or resistance to ICB therapies. Genomic factors include tumor immunogenicity, mutation/neoantigen-load (Snyder et al., 2014), increased TMB (Keenan et al., 2019), increased PD-L1 level (Havel et al., 2019), interferon gamma (FNγ) response (Ayers et al., 2017), human leukocyte antigen (HLA) diversity, deficient DNA mismatch repair (dMMR) (Zhao et al., 2019), high microsatellite instability (MSI-hi), copy-number alterations, checkpoint regulators e.g., CMTM4/6 (Mezzadra et al., 2017), up-regulation of checkpoint receptors and oncogenic signaling (Havel et al., 2019; Keenan et al., 2019). Non-genomic factors such as gut microbiome (Gopalakrishnan et al., 2018; Routy et al., 2018), metabolic pathway and the activity of lactate dehydrogenase (LDH) (Wein et al., 2018) have been shown to strongly modulate immune responses and success in ICB therapy. Some biomarkers from Table 1 and other relevant tumor tissue biomarkers for ICB therapies in breast cancer are explained below.

**TABLE 1:** A list of FDA-approved and validated ICBs targeting PD-1, PD-L1 or CTLA-4 axis with investigational biomarkers to predict an efficient patient response to the immunotherapy.

Targets mAB	ICB name (Trade name)	ICBs in cancer therapy (FDA approved) Combination with chemotherapy (#)	Biomarkers	Breast cancer (FDA unapproved)
**PD-1**	**Nivolumab** (Opdivo)	Metastatic melanoma (04/07/2014 Japan) and (13/11/2014 United States)	BRAF-V600E CD274 (PD-L1)	**Nivolumab** + short therapy of doxorubicin and cisplatin in TNBC (Voorwerk et al., 2019) + Epigenetic agent RRx-001 in TNBC (NCT02518958)
		Non-small cell lung cancer (09/10/2015) Metastatic non-small cell lung cancer (13/11/2015)		
		Metastatic renal cell carcinoma (23/11/2015)		
		Classical Hodgkin lymphoma (16/05/2016)		
		Metastatic head and neck squamous cell carcinoma (09/11/2016)		
		Advanced urolthelial carcinoma (01/02/2017)		
		Relapsed colorectal cancer (01/08/2017)	MSI-hi, dMMR	
		Advanced liver cancer (22/09/2017)		
		Metastatic small cell lung cancer (17/08/2018)		

	**Pembrolizumab** (Keytruda)	Advanced or unresectable melanoma (04/09/2014) Adjuvant treatment of stage III melanoma (15/02/2019)	BRAF-V600E	**Pembrolizumab** + a JAK2 inhibitor Ruxolitinib in TNBC (NCT03012230) + a CDK4/6 inhibitor Abemaciclib in HR + /HER2- BC (NCT02779751) + Trastuzumab in HER2 + BC (NCT02318901) + PARP inhibitor Niraparib in TNBC (NCT02657889)
		Squamous and non-squamous non-small cell lung cancer (02/10/2015) Metastatic non-small cell lung cancer (23/10/2016), Stage III non-small cell lung cancer (11/04/2019)	CD274 (PD-L1) EGFR ALK	
		Metastatic head and neck squamous cell carcinoma (04/08/2016) and first-line treatment of this cancer type (10/06/2019)		
		Adult and pediatric patients with refractory or relapsed classical Hodgkin lymphoma (14/03/2017)		
		Advanced non-small cell lung cancer and bladder cancer (09/05/2017) # and advanced bladder cancer (17/05/2017)		
		All metastatic solid tumor types (22/05/2017)	MSI-hi or dMMR	
		Stomach and gastroesophageal cancer (22/09/2017)		
		Advanced cervical cancer (12/06/2018)	CD274 (PD-L1)	
		Adult and pediatric primary mediastinal large B-cell lymphoma (13/06/2018)		
		Advanced hepatocellular carcinoma (09/11/2018)		
		Skin cancer merkel cell carcinoma (19/12/2018)		
		Metastatic small cell lung cancer (17/06/2019)		
		Advanced esophageal squamous cell cancer (30/07/2019)		
		Advanced endometrial carcinoma (27/09/2019)		
		High-risk non-muscle invasive bladder cancer (08/01/2020)		

	**Durvalumab** (Imfinzi)	Advanced bladder cancer (30/04/2017)		**Durvalumab** + Chemotherapy Taxane-anthracycline TNBC NCT02685059
		Stage III non-small cell lung cancer (16/02/2018)	CD274 (PD-L1)	

	**Cemiplimab** (Libtayo)	Advanced cutaneous squamous cell carcinoma (09/09/2018)		

**PD-L1**	**Atezolizumab** (Tecentriq)	Common type of bladder cancer (17/05/2016)		**Atezolizumab** + Trastuzumab in HER2 + BC (NCT02605915)
		Metastatic and resistant non-small cell lung cancer (17/10/2016)	Gene Signature (T-effector), ALK	
		Metastatic triple-negative breast cancer (08/03/2019) # + Nab-paclitaxel (NCT02425891)	CD274 (PD-L1)	
		Extensive-stage small cell lung cancer (18/03/2019)#		
		Metastatic non-small cell lung cancer (03/12/2019)	No EGFR or ALK aberrations	

	**Avelumab** (Bavencio)	Skin cancer merkel cell carcinoma (22/03/2017)		**Avelumab** ± CDK4/6 inhibitor Palbociclib + Tamoxifen in ER + BC (NCT03573648) + Fulvestrant in ER + /HER2- BC (NCT03147287)
		Advanced bladder cancer (08/05/2017)		
		Advanced renal cell carcinoma (14/05/2019)#	CD274 (PD-L1)	

**CTLA-4**	**Ipilimumab** (Yervoy)	Metastatic melanoma (13/11/2011)	HLA-A	

	**Tremelimumab** (Ticilimumab)	Orphan drug status for the treatment of malignant mesothelioma (20/04/2015)		

**PD-1** and **CTLA-4**	**Nivolumab** (Opdivo) and **Ipilimumab** (Yervoy)	Advanced melanoma (01/10/2015)	BRAF-V600E HLA-A	
		Advanced renal cell carcinoma (16/04/2018)		
		Relapsed or refractory colorectal cancer (10/07/2018)	MSI-hi or dMMR	

*ICB, Immune-checkpoint blockade; mAB, monoclonal antibody; BC, breast cancer; NCT, NIH clinical trial research study; BRAF-V600E, BRAF mutations of valine 600 to glutamic acid; MSI-hi, high microsatellite instability; dMMR, deficient DNA mismatch repair; HLA-A, a group of human leukocyte antigens; ALK, anaplastic lymphoma kinase and #, in combination with chemotherapy.*

Mutational landscape of the tumor and the neoantigen load are associated with increased immunogenicity which are recognized by T cells. Several studies show that high TMB correlates with enhanced ICB response rates (Keenan et al., 2019). A whole-genome sequencing (WGS) analysis of 442 patients’ tumor tissue biopsies from metastatic breast cancer revealed two fold higher TMB compared to primary breast cancer (Angus et al., 2019). They could identify 11% of patient (threshold of ≥10 mutations per Mbp) with a high TMB as a potential biomarker to identify clinically relevant subgroups for immunotherapy. Interestingly, high TMB was only associated with metastatic tissue and it was equal between breast cancer subtypes and biopsy sites.

The DNA mismatch repair (MMR) system is crucial for genomic integrity and stability and it prevents microsatellite instability (MSI). Tumors with dMMR and MSI-hi are more sensitive to anti-PD-1/PD-L1 therapies (Zhao et al., 2019). This occurs due to mutation or loss of function of DNA repair proteins. Some data show that dMMR is more frequent in early stage cancers than in metastatic cancers, which is important for the selection of the best time point for ICB therapy. Although dMMR and MSI-hi are used as predictive biomarkers for Pembrolizumab therapy of all metastatic solid tumor types, both biomarkers are rarely present in most breast tumors (Mills et al., 2018), except BRCA-deficient TNBC. BRCA1- and BRCA2-deficient breast cancers are characterized by vast genomic instability and T cell-inflamed signature. BRCA1-deficient tumors indicated high expression of PD1 and PD-L1 (Wen and Leong, 2019) and similar to TNBC, seems to have best response to ICBs, especially in combination with cytotoxic agents.

PD-L1 expression in tumor cells and TILs using immunostaining-scoring methods have been associated with response to blockade of the PD-1/PD-L1 axis (Dolled-Filhart et al., 2016; Torlakovic et al., 2020). However, there is no clear-cut between separation of responders and non-responders patients. A portion of PD-L1^–^ tumors still responds to ICB therapy and on the other hand not all PD-L1^+^ tumors are responsive to ICB therapy. PD-L1 overexpression is correlated with copy-number alteration of 9p24.1 locus containing PD-L1, PD-L2 and JAK2 (Green et al., 2010). In addition, PD-L1 protein levels and stability in tumors can be increased using inhibitors of cyclin-dependent kinases 4 and 6 (CDK4/6). It has been shown that PD-L1 protein abundance is regulated by CDK4 and cullin 3-SPOP E3 ligase via proteasome-mediated degradation (Zhang et al., 2018). To increase the ICBs efficiency in breast cancer, some clinical studies used a combination of JAK2 or CDK4/6 inhibitors with anti-PD1 therapy and evaluated the safety and efficacy of these combinational therapies in patients (NCT03012230, NCT02779751, Table 1).

Oncogenes such as mutated BRAF, EGFR and KRAS and amplified HER2 and loss of tumor suppressor genes e.g., PTEN often regulate inflammatory and immune suppressive cytokines like IL-6 and affect ICB response rates (Keenan et al., 2019). IFNγ is released by activated T cells upon recognition of tumor neoantigens and activates IFNγ-JAK-STAT-IRF1 axis in tumor cells. Alteration of this pathway affects the response to ICBs via different mechanisms such as increasing the expression levels of HLA and induction of PD-1 and PD-L1 gene expression by direct binding of IRF1 and STAT3 to their promoters (Garcia-Diaz et al., 2017; Keenan et al., 2019).

Besides TMB and the expression levels of IFNγ and PD-L1 as dynamic biomarkers for ICB therapy in breast cancer, multi-gene based assays to develop combinational ICB biomarkers are required. Similar assays e.g., Oncotype DX (Paik et al., 2004), MammaPrint (van’t Veer et al., 2002) and Prosigna (Parker et al., 2009) have been previously used for the prediction of chemo and targeted therapy benefit. The current assays such as Enzyme-Linked Immunosorbent Spot (ELISpot) for the detection of cytokine and IFNγ secreting cells show limited sensitivity in assessing tumor-specific T-cell responses. However, a new assay “Mutation-Associated Neoantigen Functional Expansion of Specific T cells” (MANAFEST) allows a sensitive measurement of antigen-specific TCR clonotypic amplifications following treatment in blood, tumor, and normal tissue of patients receiving immunotherapy (Danilova et al., 2018).

## Tumor Microenvironment Biomarkers

Extracellular matrix (ECM) changes are predicted as prognosis factors that are correlated with immunological activity. ECM dysregulation is often linked to the presence of cancer-associated fibroblasts (CAFs) expressing activated TGFβ signaling (Chakravarthy et al., 2018). The cytokine TGFβ is the major mediator of immune suppression in the tumor microenvironment and has a central role in inhibition of the both adaptive and innate immune responses during tumor progression (Batlle and Massague, 2019). Reprogramming of CAFs and anti-TGFβ therapies can enhance checkpoint blockade. A recent preclinical study has shown that the lack of response to Atezolizumab therapy was associated with TGFβ signature in fibroblasts and exclusion of CD8^+^ T-effector cells from tumor parenchyma in metastatic urothelial tumors (Mariathasan et al., 2018). Several studies revealed that combinational therapy of both TGFβ and PD-L1 resulted in synergistic anti-tumor effect in both breast and colorectal cancers (Knudson et al., 2018; Tauriello et al., 2018). A novel bifunctional anti-PD-L1/TGFβ Trap fusion protein (M7824) was tested in EMT6 and 4T1 syngeneic mouse breast cancer models. M7824 decreased TMB and promoted CD8^+^ T cell and NK cell activation (Knudson et al., 2018). In a neoadjuvant setting, M7824 is used in treating patients with stage II-III HER2^+^ Breast Cancer (NCT03620201). Besides T-cell exclusion based on TGFβ-activated stroma, Wnt-β-catenin signaling plays a role in T-cell activation and CD8^+^ T-effector cells migration by decreasing CD103^+^ dendritic cell (DC) recruitment (Spranger et al., 2015). In a melanoma model, this migration depends on the presence of DC producing CXCL10 (Spranger et al., 2017). Importantly, anti-PD-1 efficacy depends on CXCR3 activity, which is a receptor for CXCL9, CXCL10, and CXCL11 chemokines. These CXCR3 ligands are identified as positive predictive biomarkers and their induction in non-responsive mouse tumors could restore the sensitivity to anti-PD-1 (Chow et al., 2019).

Vascular endothelial growth factor A (VEGFA) and angiopoietin-2 (ANG-2) produced by endothelial cells affect antitumor immunity. In a metastatic mouse mammary tumor model (MMTV-PyMT), Schmittnaegel et al. (2017) could show that a dual inhibition of both angiogenic factors resulted in an increase of tumor antigen presentation, activation of tumor-infiltrating CD8 + T cells, and induction of endothelial PD-L1 expression through IFNγ. Simultaneous blocking of PD-L1/PD-1 signaling in this tumor model improved antitumoral activity and increased survival rate by 30% in mice (Schmittnaegel et al., 2017).

Infiltrating and tumor-associated immune cells are the major component of tumor-associated stroma with both protumor and antitumor activities. An increase in peripheral ICOS^+^CD4^+^ T cells has been also shown as a good clinical ICB responses in patients with hormone-responsive advanced breast cancer, which were treated with the anti-CTLA4 tremelimumab in combination with exemestane (Vonderheide et al., 2010).

The efficiency of ICBs is highly based on TILs and we need a better understanding of molecular determinants of TILs phenotype in tumor and tumor microenvironment. Pre-existing of immune response in tumors and localization and density of TILs are strong prognostic indicators for selection of ICB-responsive patients in different cancer types including breast cancer (Wein et al., 2018). In addition to immunohistochemistry methods to study TILs density, single cell RNA sequencing provides high-resolution to study the immune cell diversity and tumor heterogeneity related to ICB responses. Recently, Jiang et al. (2018) have developed a computational tool for dysfunctional T-cell signature, called “Tumor Immune Dysfunction and Exclusion” (TIDE). They could show that TIDE signatures predict ICB immunotherapy response in melanoma patients treated with first-line anti-PD1 or anti-CTLA4. The TIDE tool predicts only intrinsic ICB resistance and models two distinct mechanisms of tumor immune evasion: (i) T cell dysfunction in tumors with high infiltration of cytotoxic T lymphocytes (CTL), and (ii) T cell exclusion and prevention of T cell infiltration in tumors with low CTL level.

The crosstalk between cancer cells and immune cells at the primary tumor site, in the circulation and in the metastatic niche has a strong influence on cancer progression that affects patients’ response to ICBs (Saini et al., 2019). In a recent study, Wagner et al. (2019) performed large-scale mass cytometry profiling of 144 human breast tumor and 50 non-tumor tissue samples and characterized features of cancer ecosystems, inter-patient variations in tumor-associated immune cells and their associations with clinical data. They could show that PD-L1^+^ tumor-associated macrophages (TAMs) and exhausted T cells are abundant in high-grade ER^–^ and ER^+^ tumors. This single-cell mass spectrometric approach called mass cytometry (CyTOF^TM^) can be combined with immunohistochemical methods, which were used for multiplexed imaging of tumor tissues and with subcellular resolution (Giesen et al., 2014).

Reprogramming of tumor immune microenvironment presents a powerful strategy to enhance the response to anti-PD1/PD-L1 in different type of breast cancer. Recently, Voorwerk et al. (2019) showed that a short-term treatment with doxorubicin and cisplatin was able to reprogram tumor microenvironment. This caused up-regulation of inflammatory JAK-STAT and TNF-α signaling and increased the sensitivity to the Nivolumab in metastatic TNBC (Voorwerk et al., 2019).

## Future Biomarker Challenges

Scientist and healthcare professionals have gained and explained a vast knowledge about potential predictive biomarkers for ICB patient’s classification. The most promising biomarkers have been presented as proteomic and transcriptomic signatures of exosomes and CTCs, genomic analysis of ctDNA (genome instability number, specific tumor mutations, and aberrant DNA methylation signature). The next crucial step is the clinical verification of these candidate biomarkers that requires a consensus on methodological standardization of the assays and in parallel to investigate these biomarkers in large patient populations.

The interaction between tumor cells and immune cells in TME leads to the dynamic change of immunotherapy targets. This is a challenging factor for the identification of appropriate biomarkers for the selection of drug responsive patients. Biological understanding of multigene-based biomarkers and combinational strategies for ICB biomarkers will give healthcare providers the opportunity to increase the effectiveness of immune therapy in breast cancer. Advanced technologies such as single cell RNA sequencing and CyTOF-based immune profiling provide high-resolution of tumor immune microenvironment. Enhancing the drug response by remodeling of dynamic tumor ecosystem is fundamental for a successful personalized cancer therapy.

## Author Contributions

Both authors wrote the manuscript without external help. VV prepared the table.

## Conflict of Interest

The authors declare that the research was conducted in the absence of any commercial or financial relationships that could be construed as a potential conflict of interest.
